# Nurses' Knowledge and Attitudes Toward Pain Assessment and Management: A Cross-Sectional Study

**DOI:** 10.1155/nrp/6646998

**Published:** 2025-06-26

**Authors:** Amal Wanis Alanizi, Wejdan Shaqiqi, Lerma C. Cabaldo, Razan Mohammed Awaji, Reham Abdulkarem Alotaibi, Asma Mohamed Alanazi

**Affiliations:** ^1^College of Nursing, King Saud Bin Abdulaziz University for Health Sciences, Riyadh, Riyadh, Saudi Arabia; ^2^King Abdullah International Medical Research Center, Riyadh, Riyadh, Saudi Arabia

**Keywords:** attitude, knowledge, nurses, pain, pain assessment, pain management

## Abstract

**Background:** Pain remains a complex and multifaceted challenge in healthcare, affecting patient outcomes, quality of life, and healthcare costs. Significant gaps in knowledge and attitudes among nurses can hinder effective pain management, patient outcomes, and satisfaction.

**Purpose:** This study assesses nurses' knowledge and attitudes regarding pain assessment and management.

**Methods:** A quantitative descriptive cross-sectional design was employed. Convenience sampling was used to recruit 161 registered nurses from various wards at a hospital in Riyadh, Saudi Arabia, between January and April 2024. Data were collected using the Knowledge and Attitude Survey Regarding Pain (KASRP) and analyzed using descriptive statistics, one-way ANOVA, and independent *t*-tests.

**Results:** The nurses' knowledge and attitudes regarding pain were moderate (*M* = 25.62, SD ± 6.4). Less than a quarter of participants demonstrated a good level according to the KASRP (22.36%). The score was greater among nurses working in surgical, emergency, and hemodialysis units (*F* = 2.47, *p*=0.03), those with good perceived competency levels in pain management (*t* = 3.41, *p* < 0.001) and knew about the availability of pain management protocols in the unit (*t* = 2.81, *p*=0.003).

**Conclusion:** The findings underscore the need for enhanced educational strategies to improve nurses' knowledge of pain assessment and pharmacological interventions, and to address misconceptions about opioid use and opioid dependency, as well as the ethical implications of placebo use. Continued professional development and the implementation of standardized protocols are recommended to improve nursing practice in this area.

## 1. Introduction

Pain is a widespread healthcare issue that affects millions of people worldwide and is a primary reason leading patients to seek medical care [1]. The International Association for the Study of Pain (IASP) defines pain as “an unpleasant sensory and emotional experience associated with actual or potential tissue damage or described in terms of such damage” [2]. According to IASP, pain is classified as acute or chronic, and each has a different management approach [2]. Acute pain usually lasts for a short time, develops from an injury or illness, and is mostly managed with proper treatment. On the other hand, chronic pain lasts for 3 months or more, extends beyond the healing period, and may result from physiological and psychological factors.

The prevalence of pain is increasing worldwide, with the IASP estimating that one in five people experience pain, and one in 10 are diagnosed with chronic pain [3, 4]. A global study involving 146 countries and 1.6 million respondents found that the prevalence of pain increased from 26.3% to 32.1% between 2009 and 2021 [5]. Pain affects individuals in various healthcare settings, including hospitals, outpatient clinics, and long-term care facilities. A study by Wu et al. [6] revealed that 69.5% of hospitalized patients reported experiencing pain, with 43% suffering from severe pain.

Pain not only affects physical health but also has significant psychological and emotional consequences [7]. Studies have linked poorly managed pain to post-traumatic stress disorder (PTSD), depression, anxiety, and sleep disturbances [8, 9]. Additionally, prolonged pain can contribute to cognitive dysfunction, reduced concentration, mental confusion, and even delirium [9]. Physiologically, chronic pain can impair immune function, prolong mechanical ventilation, and lead to hemodynamic instability [10]. Patients with poorly controlled pain often have extended hospital stays, increased morbidity, and higher healthcare costs [11]. Studies indicate that well-managed pain contributes to faster recovery, improved sleep quality, enhanced mobility, and overall higher patient satisfaction [11].

Nurses play a central role in pain assessment and management as they are the primary healthcare providers responsible for direct patient care [12]. Among all healthcare professionals, nurses spend the most time with patients, making their knowledge and attitudes toward pain critical for ensuring timely and effective pain relief [12]. Their ability to assess pain accurately, administer appropriate analgesics, and advocate for necessary interventions significantly impacts patient outcomes [13]. Therefore, understanding nurses' knowledge and attitudes toward pain and addressing identified gaps are essential to prevent inadequate pain control that can lead to unnecessary patient suffering, prolonged hospital stays, and increased healthcare costs [14].

In Saudi Arabia, a significant portion of the nursing workforce comprises foreign-trained nurses. According to the latest data, international nurses comprise about 60% of the total number of nurses and are mostly Indian, Filipino, and Malaysian [15]. These nurses come from diverse educational backgrounds. In addition, language and cultural barriers—such as religious beliefs, societal norms, and traditional healing practices—can impair effective communication with patients and influence how individuals perceive, express, and manage pain-related symptoms [16]. Some cultures emphasize emotional restraint and may underreport pain, while others expect healthcare providers to proactively manage pain regardless of patient complaints [17]. Understanding how these cultural variations shape nurses' attitudes is critical to ensuring standardized, high-quality pain assessment and management across different healthcare settings. In Saudi Arabia, where the nursing workforce is predominantly foreign-trained, this becomes even more essential to ensure patient-centered care and minimizing disparities in pain treatment. Cultural beliefs in Saudi Arabia, such as valuing stoicism and underreporting pain, can impact both patients' expression of pain and nurses' interpretation and response [18]. Patient–nurse interactions are thus influenced not only by clinical knowledge but also by culturally embedded expectations regarding pain behavior from both the patient and the nurse. These cultural dynamics may partially explain observed variations in nurses' knowledge and practices related to pain management [17]. Recognizing these factors is essential to interpreting the findings and comparing them in a global context, where cultural norms surrounding pain and healthcare communication can differ substantially [19].

Studies have highlighted variations in nurses' knowledge and attitudes regarding pain across different countries. A study in Ghana demonstrated that the majority of surgical nurses had moderate knowledge and negative attitudes [20]. A study in hospitals in low-income areas of China found widespread deficiencies in nurses' pain management knowledge, with none of the nurses achieving an adequate knowledge score [21]. Similarly, a study in Palestine reported that ICU nurses faced multiple barriers to effective pain management despite moderate knowledge levels [22]. In addition, a systematic review that included 18 studies with 7942 nurses highlighted that the nurses' knowledge and attitudes was 52.9%, which is lower than the recommended percentage of 80% [23]. These findings emphasize the global challenges in ensuring adequate pain knowledge and attitudes among nursing staff, especially in different healthcare contexts, and the need for continuous assessment. Cultural differences in pain perception and treatment further complicate the standardization of pain management practices [19].

There remains a scarcity of research examining the knowledge and attitudes toward pain assessment and management in Saudi Arabia. A few studies were conducted in Saudi Arabia on nurses, and they have identified critical deficiencies in nurses' understanding and attitudes toward pain assessment and management [24–26]. None of these studies were conducted in a military hospital, and only a few were conducted in Riyadh [27], which is the capital of Saudi Arabia. Therefore, this study aims to assess nurses' knowledge and attitudes toward pain assessment and management in a military hospital in Riyadh, Saudi Arabia. Additionally, it explores their association with the participants' demographic characteristics, such as nationality and working unit, and with their self-perceived competency, prior training, and protocol awareness.

## 2. Methods

### 2.1. Design and Setting

This study followed the Strengthening the Reporting of Observational Studies in Epidemiology (STROBE) checklist. A quantitative descriptive cross-sectional design was used. Data were collected at a tertiary-care military hospital in Riyadh, Saudi Arabia. It is considered one of the most comprehensive healthcare facilities in Saudi Arabia, with a total bed capacity of 2151.

### 2.2. Participants, Sample, and Sampling

A convenient sampling technique was used to recruit the participants. The inclusion criteria were registered nurses from various inpatient care units and with at least 6 months of experience. Nurses from the outpatient department and those in administrative roles were excluded. Nurses who were newly hired with less than 6 months of experience, those undergoing a preparatory program, interns, student nurses, and nurses working in the pain management team were excluded.

The required sample size was calculated using G^∗^Power software 3.1. With a target population of 800, and considering an 80% confidence level, an effect size of 0.15, a significance threshold of 0.05, and 10 predictors, and the minimum estimated sample size was 118. The number of participants recruited for the study was 161.

### 2.3. Measurement

Data were collected using a questionnaire composed of two sections. The first section consisted of demographic data, comprising 10 questions on the demographic, educational, and clinical information of the participants. This included age, gender, years of experience, qualifications, and assigned area.

The second section was the Knowledge and Attitude Survey Regarding Pain (KASRP) developed in 1987 and revised in 2014 [28]. It consists of 41 items, including 22 true/false questions, 15 multiple-choice questions, and four case studies. The KASRP is a reliable and validated tool that has been widely used among nurses and other professionals in various healthcare settings and countries including Saudi Arabia. Reliability values for the KASRP have been established in previous studies, with test–retest reliability (*r* > 0.80) and internal consistency reliability (*α* > 0.70) [28]. Furthermore, it has an established content validity by a panel of pain experts, based on pain management guidelines from the American Pain Society, the World Health Organization, and the Agency for Health Care Policy and Research [28]. Construct validity has been determined by comparing the scores of nurses at various levels of expertise, such as students, new graduates, oncology nurses, graduate students, and senior pain experts [28]. In the current study, the KASRP demonstrated Cronbach's alpha of 0.81, indicating good internal consistency [29].

The original English version of the KASRP was used without translation, as English is the primary language of nursing education and practice in Saudi Arabia. Additionally, no modification was required as the original tool has been used in previous studies conducted in Saudi Arabia. The authors recommend reporting the KASRP score in total and not distinguishing between knowledge and attitude as many items measure both variables. For scoring, each question has only one correct response and is marked as one, and wrong answers are zero. The total score is then converted into a percentage, with < 50% graded as poor, 50%–75% as fair, and > 75% as good. Then, the mean is calculated, ranging from 0 to 41. A higher score indicates better knowledge and a more positive attitude.

### 2.4. Data Collection

The data were collected from January to April of 2024 using a self-reported web survey developed by Microsoft Forms. The web survey was sent to the email of nursing staff and the nurse managers working in a government hospital in Riyadh, Saudi Arabia. To enhance recruitment, the principal investigator visited the units, invited the targeted nurses to participate, and answered any questions.

### 2.5. Data Analysis

Data were managed and analyzed using the Statistical Package for Social Sciences (SPSS, version 22). Nurses' categorized KASRP scores are presented using descriptive statistics, such as percentages and frequencies. An independent *t*-test and one-way ANOVA were employed to determine the mean differences in KASRP according to sample demographic characteristics [30]. The significance level was set at *p* < 0.05.

## 3. Ethical Considerations

The researchers adhered to all ethical considerations in conducting the present study. Ethical approval was obtained from the Institutional Review Board (IRB) of King Abdullah International Medical Research Center (KAIMRC) with ethical approval number SP23R/193/08. All data were protected by password and were accessible by the research team only. The questionnaire was kept securely with the researcher in a locked facility to preserve the data. An electronic consent form was obtained from all participants before completing the questionnaire, and anonymity was maintained throughout the study. The consent form explained the research aim, that no personal risk was associated with participation, and informed participants of the voluntary nature of the study and their right to withdraw at any time.

## 4. Results

Table 1 shows the participants' demographic data. Most participants were 35 years old or younger (54%), were female (83.9%), were BSc holders (68.9%), and had 6–10 years of experience (35.4%). The majority of nurses had received a formal course in the past 5 years (93.1%). Most of the nurses knew about the pain management protocol (80.7%) and perceived themselves competent in pain management (71.4%).

The participants' KASRP mean score was moderate (*M* = 25.62, SD ± 6.4) and approximately a quarter of the participants demonstrated a good level of KASRP (22.36%) (see Table 2). The frequency and rank of correctly answered items are shown in Table 3. Out of the 41 items, 11 scored above 75% (26.8%), and 16 items scored below 50% (39%). The highest-scoring items were the most accurate judge of the pain intensity is the patient (90.7%), the meaning of “equinalgesia” term (85.7%), and morphine is considered the drug of choice for the treatment of prolonged moderate to severe pain for cancer patients (85.7%). The least scored items were placebo being a useful test to determine if the pain is real (24.8%), after an initial dose of opioid analgesic being given, subsequent doses should be adjusted in accordance with the individual patient's response (21.1%), and children less than 11 years cannot reliably report pain (15.5%).

Table 4 presents the significant differences in the participants' KASRP levels according to their information. The KASRP score was significantly associated with the unit in which participants were based. Nurses working in surgical (*M* = 28.25, SD ± 6.40), emergency (*M* = 27.00, SD ± 4.76), and hemodialysis units (*M* = 26.73, SD ± 7.22) obtained significantly higher mean score in the KASRP compared to those in cardiac, intensive care, and medical units (*F = *2.47, *p*=0.03). Moreover, participants who had taken pain management courses within the past 5 years (*M* = 25.76, SD ± 6.42) scored higher mean compared to their counterparts (*M* = 23.91, SD ± 5.98), but the difference was not statistically significant (*t* = 2.08, *p*=0.05). Nurses who rated their competency in pain management as good (*M* = 26.83, SD ± 5.41) had a higher mean compared to their counterparts (*t* = 3.41, *p* < 0.0001). Lastly, those who reported the availability of pain management protocols in their unit (*M* = 26.30, SD ± 6.34) rated higher in KASRP compared to those who did not know about such protocols (*t* = 2.81, *p*=0.003).

## 5. Discussion

This study aimed to evaluate the knowledge and attitudes of nursing staff in regard to pain assessment and management. The findings reveal both strengths and areas that need improvement in pain assessment and management among the participants, offering valuable insights into the current state of nurses' knowledge and attitudes. The majority of nurses demonstrated a low to moderate level of knowledge and attitude regarding pain. Similar results were found in both national [24, 25] and international [31] studies. They reported low to moderate performance in knowledge and attitude among healthcare professionals, including nurses. In these studies, the majority of nurses exhibited inadequate knowledge regarding pain, particularly concerning pharmacological interventions. Thapa et al. [31] noted that healthcare professionals were considered to have adequate knowledge if they scored 80% or above, however many fell short of this benchmark, similar to the findings in the current study. Similarly, Jamal et al. [25] found that 48.2% of nurses had poor knowledge and only 7.1% had good knowledge, despite the availability of pain assessment tools and protocols.

Most of the poorly answered items were related to pharmacological interventions, including the appropriate selection, dosage, and rotation of opioid classes. For instance, more than half of the nurses failed to recognize the signs and symptoms of opioid withdrawal. Additionally, many did not understand that subsequent opioid doses should be adjusted based on the patient's response to the initial doses. Similar gaps have been identified in international studies [23] and in Saudi Arabia. Al Mukalas et al. [32] found that most emergency nurses in Najran, Saudi Arabia, had low pharmacological knowledge that affected correct analgesic selection and dosing. Likewise, Jamal et al. [25] reported that nurses in intensive care units face major pain management challenges due to inadequate opioid administration and dose adjustments. Pharmacological interventions play a crucial role in effective pain treatment, however the findings of our studies and others have shown that a lack of adequate knowledge regarding opioid addiction, tolerance, and side effects contributes to the under prescription and inadequate administration of analgesics, leading to ineffective pain control [33–35]. Additionally, many nurses lack an understanding of opioid pharmacokinetics, appropriate dosage titration, and risk management strategies for adverse effects [16]. Fear of opioid-induced respiratory depression often results in unnecessary restrictions on opioid use, limiting patient access to effective pain relief [34]. Limited training in pain medication management has been noted as a contributing factor to these deficiencies. A study conducted in Saudi Arabia found that 70% of nurses lacked sufficient training in opioid administration, proper dosage adjustments, and side effect management, emphasizing the need to enhance pharmacological training [24].

Nurses had misconceptions regarding pain which can result in ineffective pain assessment and management. More than half of nurses in this study failed to recognize that patients can be distracted or sleep even with severe pain, a misconception also highlighted in previous studies from Saudi Arabia, Nepal, and Vietnam, where nurses relied on patients' behaviors rather than self-reported pain levels [24, 31, 36]. Similarly, two-thirds of nurses believed that vital signs are always reliable indicators of the intensity of a patient's pain, despite evidence that pain is a personal experience best assessed through self-report [36].

Additionally, this study found that several nurses believed patients should endure pain before using opioids, which can lead to unnecessary suffering and delayed analgesics administration. These concerns were reported in studies conducted in Saudi Arabia and Nepal, where nurses expressed their fear of opioid dependence and addiction that affected their decision to administer appropriate pain medications [24, 37]. Another reported concern in this study was the inappropriate use of a placebo to determine if the patients' pain was real, despite the unethical implications. Similarly, other studies have reported these misconceptions and their serious implications, as they can lead to inadequate pain relief, prolonged hospitalization, and reduced patient satisfaction [37].

In this study, no significant difference was found between local and expatriate nurses. Nevertheless, cultural factors may have influenced nurses' knowledge and attitudes toward pain assessment and management. In Saudi Arabia, societal norms often encourage emotional restraint and the underreporting of pain [18]. Local nurses and those who share similar cultural beliefs may internalize these norms, which can impact their assessment and management of pain [16, 17]. Conversely, expatriate nurses who are unfamiliar with local cultural beliefs—combined with potential language barriers—may face challenges in pain management, often prioritizing observable signs over patient self-report [38]. Understanding this cultural context is crucial, as it underscores that nurses' knowledge and practices are shaped not only by education and experience but also by prevailing cultural values [16]. This should be considered when comparing results internationally, as norms surrounding pain expression and patient-provider communication can vary widely. Such dynamics highlight the need for culturally sensitive training that helps nurses recognize nonverbal cues and supports patient advocacy across diverse cultural settings.

In this study, there were significant differences in nurses' knowledge and attitude toward pain according to units, competency level, and knowledge of pain management protocol. Nurses working in surgical, emergency, and hemodialysis units exhibited higher knowledge and attitude, whereas those in cardiac, intensive care, and medical units had lower understanding. This variation across different hospital wards is further supported by previous findings [24]. This may be attributed to the frequent exposure to acute pain cases in emergency and surgical settings compared to other units. Jamal et al. [25] also found that critical care nurses with more exposure to pain scenarios had significantly better knowledge and attitude. Additionally, Al Mukalas et al. [32] reported that nurses who regularly manage patients experiencing pain, especially in emergency settings, had higher pain knowledge and fewer misconceptions. Continuous exposure to pain management cases can enhance competency, reinforcing the importance of involving hands-on and clinical cases scenarios in the educational programs [32, 39]. It is also possible that nurses in surgical, emergency, and hemodialysis units receive more frequent pain management training and are more familiar with standardized pain protocols, which may contribute to their higher knowledge and attitudes.

Despite the presence of pain management protocols, nearly a quarter of the nurses in this study were unaware of their availability, a concern echoed in [26], where lack of access to standardized guidelines was cited as a major barrier to effective pain management. Thapa et al. [31] also indicate that a lack of institutional policy and guidelines regarding pain management further exacerbates the issue associated with knowledge deficit. This gap may be attributed to the lack of ongoing training and guidance and a failure to implement such protocols. As our study indicated, nurses who were aware of the pain management protocol in the hospital had significantly higher KASRP. This highlights the need for better dissemination and implementation of pain management protocols across healthcare institutions.

Nurses who rated themselves as competent demonstrated higher KASRP scores, whereas those with lower knowledge acknowledged their limitations, which aligns with the findings from the authors in [13, 27]. These studies similarly found that nurses who rated themselves as competent demonstrated significantly higher knowledge and positive attitudes toward pain management. This emphasizes the critical role of self-perceived competency in pain management and suggests that fostering self-assessment and competency-based training can enhance knowledge retention and improve patient care [13]. Moreover, a study conducted in Vietnam found that nurses who self-assessed their competency as high showed better knowledge and attitude toward pain assessment and management [36]. Similarly, in Saudi Arabia, Al-Sayaghi et al. [24] reported that nurses with strong self-perceived competency were more likely to participate in continuous learning and apply pain management protocols effectively. This supports the notion that self-assessment can be a valuable tool to recognize educational gaps and improve professional development programs [24, 36]. A structured competency framework incorporating self-assessment tools, mentorship programs, and case-based learning can further strengthen nurses' proficiency in pain management.

Lastly, nurses in this study who attended formal pain management courses within the past 5 years demonstrated superior knowledge and attitudes; however, the difference was not significant. The numbers between the two groups vary significantly, making it difficult to draw a solid conclusion. Nevertheless, the association between formal pain management training and improved knowledge is well-documented [32, 40]. Additionally, Al Mukalas et al. [32] and Al-Sayaghi et al. [24] highlighted the positive impact of continued professional education on nurses' pain assessment and management competencies. The finding that many nurses still demonstrated only moderate knowledge and attitudes despite high attendance in pain management courses suggests a need to critically assess the content, teaching strategies, and long-term effectiveness of the current educational programs. Future research should explore how pain management training can be optimized to produce more substantial and lasting improvements.

## 6. Limitations and Future Research

This study's limitations include its cross-sectional design, which restricts the ability to infer causality. The small sample size, reliance on convenience sampling, and single-site design may limit the generalizability of the findings. Although our study is one of the few conducted in a tertiary-care military hospital, this may limit the generalizability of the findings to civilian hospitals. Military hospitals in Saudi Arabia often have standardized training programs and structured protocols [41], which could influence nurses' knowledge and attitudes toward pain management. Additionally, compared to military settings, patient populations in civilian hospitals are often more diverse in terms of age, health status, and cultural background. Therefore, caution should be exercised when extrapolating these findings to nonmilitary healthcare environments. Additionally, self-reported data on pain management skills and knowledge may introduce bias, including possible social desirability bias.

Future studies are recommended to use random sampling across different hospitals to improve generalizability and could benefit from using objective measures to validate self-reported responses. Longitudinal studies are recommended to assess how nurses' knowledge and attitudes toward pain management change over time following targeted educational interventions or clinical practice exposure.

## 7. Implications

This study highlighted several significant findings that call for educational and institutional implications locally and globally, given that 67.7% of the nurses who participated in this study were non-Saudi from different nationalities and backgrounds. The findings underscore the need for continued training programs of pain assessment, diagnosis, and treatment options that utilize theoretical and clinical strategies. It should involve pain pharmacology materials, correct any misconceptions, and address identified nurses' educational needs through assessment and self-assessment competency. As supported by the existing evidence, ongoing professional development is crucial for maintaining up-to-date knowledge and skills in pain management [42–44]. The promoting of self-assessment competency can help identify areas for improvement and enhance their skills in pain management, as nurses are aware of their limitations. Furthermore, there is a need for department-specific training modules tailored to the unique demands of different hospital units. Periodic pain education that accounts for cultural diversity, varied professional experiences, and different healthcare settings designed for an internationally diverse workforce can promote more unified, evidence-based practices across healthcare settings, ensuring optimal pain management for patients worldwide. Lastly, the effectiveness and the long-term impact of educational interventions on pain management knowledge, attitude, and practices should be evaluated.

The study suggests incorporating pain educational content in undergraduate nursing curricula as a vital and early step to sustain long-term competency in pain management. For healthcare policymakers, establishing and implementing pain management protocols and guidelines in the institution is essential to guide nurses' practice and ensure consistent, high-quality patient care. Future research should focus on longitudinal studies to assess the nurses' pain knowledge and attitude, and explore these practices in diverse settings. Additionally, qualitative research could provide deeper insights into the cultural barriers and facilitators of effective pain management in clinical practice.

## 8. Conclusion

While this study reveals some positive aspects of pain knowledge and attitude among nursing staff, it also reveals significant gaps in pain assessment and management. While the overall knowledge and attitude of nurses about pain management was fair, it was not satisfactory in terms of ensuring effective practice. This is concerning, especially given that many participants demonstrated limited understanding in key areas, particularly regarding pharmacology related to pain management. Addressing these gaps requires a multifaceted approach, including curriculum reform, continuous training, and institutional policy enhancements. By enhancing knowledge and attitudes toward pain management, nurses can better meet the needs of their patients and improve overall care outcomes.

## Figures and Tables

**Table 1:** The participants' demographic data (*N* = 161).

Demographic data	*N* (%)
Age	
30 years and below	44 (27.3)
31–35 years	43 (26.7)
36–40 years	51 (31.7)
41 years and above	23 (14.3)
Gender	
Female	135 (83.9)
Male	26 (16.1)
Nationality	
Saudi	52 (32.3)
Non-Saudi	109 (67.7)
Marital status	
Single	60 (37.3)
Married	87 (54)
Others (divorced/widowed/separated)	14 (8.7)
Years of experience	
5 years and below	40 (24.8)
6–10 years	57 (35.4)
11–15 years	39 (24.2)
15 years and above	25 (15.5)
Qualification	
Diploma	27 (16.8)
BSc nursing	111 (68.9)
MSc or higher	23 (14.3)
Unit/ward	
Critical care unit	23 (14.3)
Cardiac care unit	25 (15.5)
Emergency care unit	24 (14.9)
Medical care unit	30 (18.6)
Surgical care unit	28 (17.4)
Hemodialysis services unit	15 (9.3)
Others	16 (9.9)
Receive/attend pain management content (workshops, lectures, seminars)	
Yes	150 (93.1)
No	11 (6.8)
Aware of the pain management protocol in the hospital	
Yes	130 (80.7)
No	5 (3.1)
Don't know	26 (16.1)
Perceiving yourself competent in pain management	
Good	115 (71.4)
Fair	46 (28.6)

**Table 2:** The knowledge and attitude regarding pain (*N* = 161).

Variables	Poor *N* (%)	Fair *N* (%)	Good *N* (%)	Mean (SD)	Range
Knowledge and attitude regarding pain	33 (20.50%)	92 (57.14%)	36 (22.36%)	25.62 (6.4)	0–41

*Note:* Poor < 50%; fair 50%–75%; good > 75%.

**Table 3:** Frequency of the correct response to knowledge and attitude regarding pain (*N* = 161).

Rank	Item no	Items	*N* (%)
*Items with good score*			
1	31	The most accurate judge of the intensity of the patient's pain is (the patient)	146 (90.7)
2	21	The term “equinalgesia” means approximately equal analgesia and is used when referring to the doses of various analgesics that provide approximately the same amount of pain relief (T)	138 (85.7)
2	25	Which of the following analgesic medications is considered the drug of choice for the treatment of prolonged moderate to severe pain for cancer patients? (morphine)	138 (85.7)
3	23	The recommended route administration of opioid analgesics for patients with brief, severe pain of sudden onset such as trauma or postoperative pain is: (intravenous)	137 (85.1)
3	40	Patient B. On the patient's record, you must mark his pain on the scale below. Circle the number that represents your assessment of Robert's pain: (8)	137 (85.1)
4	20	Narcotic/opioid addiction is defined as a chronic neurobiologic disease, characterized by behaviors that include one more of the following: impaired control over drug use, compulsive use, continued use despite harm, and craving (T)	133 (82.6)
5	13	Patients' spiritual beliefs may lead them to think pain and suffering are necessary (T)	132 (82)
6	2	Because their nervous system in underdeveloped children under 2 years of age have decreased pain sensitivity and limited memory of painful experiences (F)	130 (80.7)
7	7	Combining analgesics that work by different mechanisms (e.g., combining an NSAID with an opioid) may result in better pain control with fewer side effects than using a single analgesic agent (T)	128 (79.5)
8	29	The most likely reason a patient with pain would request increased doses of pain medication is: (The patient is experiencing increased pain)	123 (76.4)
9	22	Sedation assessment is recommended during opioid pain management because excessive sedation precedes opioid-induced respiratory depression (T)	122 (75.8)

*Items with fair score*			
10	30	Which of the following is useful for treatment of cancer pain? (All of the above)	119 (73.9)
11	32	Which of the following describes the best approach for cultural considerations in caring for patients in pain: (The patient should be individually assessed to determine cultural influences)	114 (70.8)
12	38	Patient A. On the patient's record, you must mark his pain on the scale below. Write the number that represents your assessment of Andrew's pain. (8)	112 (70)
13	5	Aspirin and other nonsteroidal anti-inflammatory agents are not effective analgesics for painful bone metastases (F)	106 (65.8)
14	34	The time to peak effect for morphine given IV is: (15 min)	105 (65.2)
15	19	Benzodiazepines are not effective pain relievers and are rarely recommended as part of analgesics regiment (T)	103 (64)
16	37	Which statement is true regarding opioid-induced respiratory depression: (Obstructive sleep apnea is an important risk factor)	102 (63.4)
17	35	The time to peak effect for morphine given orally is: (1-2 h)	100 (62.1)
18	8	The usual duration of analgesia of 1-2 mg morphine IV is 4-5 h (F)	95 (59)
19	17	If the source of the patient's pain is unknown, opioids should not be used during the pain evaluation period, as this could mask the ability to correctly diagnose the cause of pain (F)	92 (57.1)
20	9	Opioids should not be used in patients with a history of substance abuse (F)	90 (55.9)
21	33	How likely is it that patients who develop pain already have an alcohol and/or drug abuse problem? (5%–15%)	84 (52.2)
22	27	Analgesics for postoperative pain should initially be given (around the clock on a fixed schedule)	83 (51.6)
23	28	A patient with persistent cancer pain has been receiving daily opioid analgesics for 2 months. Yesterday the patient was receiving morphine 200 mg/hour intravenously. Today he has been receiving 250 mg/hour intravenously. The likelihood of the patient developing clinically significant respiratory depression in the absence of new comorbidity is: (Less than 1%)	83 (51.6)

*Items with poor score*			
24	3	Patients who can be distracted from pain usually do not have severe pain (F)	78 (48.4)
24	6	Respiratory depression rarely occurs in patients who have been receiving stable doses of opioids over a period of months (T)	78 (48.4)
25	4	Patients may sleep in spite of severe pain (T)	71 (44.1)
25	36	Following abrupt discontinuation of an opioid, physical dependence is manifested by the following: (Sweating, yawning, diarrhea, and agitation with patients when the opioid is abruptly discontinued)	71 (44.1)
26	39	Your assessment, above, is made 2 h after he received morphine 2 mg IV. Half hourly pain ratings following the injection ranged from 6 to 8, and he had no clinically significant respiratory depression, sedation, or other untoward side effects. He has identified 2/10 as an acceptable level of pain relief. His physician's order for analgesia is “morphine IV 1–3 mg q1h prn pain relief.” Check the action you will take at this time: (Administer morphine 3 mg IV now)	66 (41)
27	23	The recommended route of administration of opioid analgesics for patients with persistent cancer-related pain is: (Oral)	64 (39.8)
28	18	Anticonvulsant drugs such as gapapentin (neurontin) produce optimal pain relief after single dose (F)	63 (39.1)
29	11	Patients should be encouraged to endure as much pain as possible before using an opioid (F)	60 (37.3)
30	26	A 30-mg dose of oral morphine is approximately equivalent to: (Morphine 10 mg IV)	56 (34.8)
30	1	Vital signs are always reliable indicators of the intensity of a patient's pain (F)	56 (34.8)
31	10	Elderly patients cannot tolerate opioids for pain relief (F)	52 (32.3)
32	16	Vicodin (hydorcodone 5 mg + acetaminophen 300 mg) PO is approximately equal to 5–10 mg of morphine PO (T)	49 (30.4)
33	41	Your assessment, above, is made 2 hours after he received morphine 2 mg IV. Half hourly pain ratings following the injection ranged from 6 to 8 and he had no clinically significant respiratory depression, sedation, or other untoward side effects. He has identified 2/10 as an acceptable level of pain relief. His physician's order for analgesia is “morphine IV 1–3 mg q1h prn pain relief.” Check the action you will take at this time. (Administer morphine 3 mg IV now)	46 (28.6)
34	15	Giving patients sterile water by injection (placebo) is a useful test to determine if the pain is real (F)	40 (24.8)
35	14	After an initial dose of opioid analgesic is given, subsequent doses should be adjusted in accordance with the individual patient's response (T)	34 (21.1)
36	12	Children less than 11 years old cannot reliably report pain so clinicians should rely solely on the parent's assessment of the child's pain intensity (F)	25 (15.5)

*Note:* Poor < 50%; fair 50%–75%; good > 75%.

**Table 4:** Analysis of variance test for pain management knowledge and attitude against participants' sociodemographics.

Variable	Number	KASRP			95% CI	
		M (SD)	*t* or *f*	*p*	LPCI	UPCI
Nationality						
Saudi	52	26.30 (4.90)	0.939	0.175	−1.119	3.147
Non-Saudi	109	25.29 (7.01)				
Unit/ward						
Critical care unit	23	24.91 (5.98)	2.47^∗^	0.03	0.600	0.649
Cardiac care unit	25	22.32 (6.44)				
Emergency care unit	24	27.00 (4.76)				
Medical care unit	30	24.37 (5.65)				
Surgical care unit	28	28.25 (6.40)				
Hemodialysis services unit	15	26.73 (7.22)				
Others	16	26.44 (7.76)				
Competency						
Average	115	22.61 (7.67)	3.41^∗∗∗^	< 0.001	2.103	6.331
Good	46	26.83 (5.41)				
Received/attended pain management content in the last 5 years						
Yes	150	25.76 (6.42)	2.08	0.05	−0.779	7.023
No	15	22.63 (4.67)				
Aware of the pain management protocol in the hospital						
Yes	130	26.30 (6.34)	2.81^∗∗^	0.003	1.049	6.002
No/don't know	31	22.77 (5.95)				

^∗^ *p* < 0.05.

^∗∗^ *p* < 0.01.

^∗∗∗^ *p* < 0.001.

## Data Availability

The data that support the findings of this study are available on request from the corresponding author. The data are not publicly available due to privacy or ethical restrictions.
